# Investigation of surface topography and stiffness on adhesion and neurites extension of PC12 cells on crosslinked silica aerogel substrates

**DOI:** 10.1371/journal.pone.0185978

**Published:** 2017-10-19

**Authors:** Kyle J. Lynch, Omar Skalli, Firouzeh Sabri

**Affiliations:** 1 Dept. of Physics and Materials Science, University of Memphis, Memphis, Tennessee, United States of America; 2 Dept. of Biological Sciences, University of Memphis, Memphis, Tennessee, United States of America; The University of Akron, UNITED STATES

## Abstract

Fundamental understanding and characterization of neural response to substrate topography is essential in the development of next generation biomaterials for nerve repair. Aerogels are a new class of materials with great potential as a biomaterial. In this work, we examine the extension of neurites by PC12 cells plated on matrigel-coated and collagen-coated mesoporous aerogel surfaces. We have successfully established the methodology for adhesion and growth of PC12 cells on polyurea crosslinked silica aerogels. Additionally, we have quantified neurite behaviors and compared their response on aerogel substrates with their behavior on tissue culture (TC) plastic, and polydimethylsiloxane (PDMS). We found that, on average, PC12 cells extend longer neurites on crosslinked silica aerogels than on tissue culture plastic, and, that the average number of neurites per cluster is lower on aerogels than on tissue culture plastic. Aerogels are an attractive candidate for future development of smart neural implants and the work presented here creates a platform for future work with this class of materials as a substrate for bioelectronic interfacing.

## Introduction

One of the first steps towards the development of modern biomaterials to engineer neuronal scaffolds is to characterize the biophysical interactions between neuronal cell and the surface of the material. Recent studies have shown that substrates with micro- [1, 2] and nanostructured [3, 4] surfaces provide topographical cues that can positively influence cellular response in tissue culture systems. More specifically, mechanical properties, such as stiffness [1–12], and topographical features of the substrate onto which cells attach influence parameters including cell survival, proliferation, adhesion, differentiation and metabolism [1–5]. Consequently, topography and mechanical properties of the substrate onto which cells are attached can be engineered to control and regulate specific cellular functions and activities [13]. Studies have also shown that the level of cytocompatibility and cell-material interaction may be modulated not only by means of surface roughness and stiffness [1–14], but also by biochemical stimulation via the release of biological growth factors [15], and electrical stimulation [16,17]. The ability to precisely control the adhesion, proliferation, and growth rate of cells and more specifically neurons, to a substrate is an important stage of creating and utilizing novel materials for tissue engineering applications [17]. The design and successful implementation of smart electrically active implants is currently limited by the availability of biostable and biocompatible substrate materials that can also tolerate all the required processing steps involved in fabricating suitable bioelectronic interfaces [17].

Recent studies have also demonstrated the importance of the porosity of the substrate on the adhesion, proliferation, and differentiation of various cell types including human mesenchymal stem cells [18], neurons [19], mouse 3T3 fibroblasts, human vascular endothelial cells (HUVECs), mouse neuroblastoma cells (N2A) and immortalized human cortical neuronal cells (HCN1A) [20]. These studies have demonstrated the notion that cells sense nanoscopic and microscopic topographical features of the substratum onto which they are supported by and that they react differently to pore of different sizes. Overall, these studies revealed a preference for nanometer-sized pore sizes relative to micrometer sized pores with respect to stronger cell-substratum adhesion and faster growth rate [18]. One type of mesoporous material with great potential as a biomedical material is represented by polyurea crosslinked silica aerogels [21–27]. These are light-weight mesoporous materials with tunable surface and bulk properties which, when chemically crosslinked, offer a unique combination of mechanical strength and a rich 3-D surface topography [22]. In general, aerogels are known for their light weight, extreme low density, and high degree of porosity (over 99% open pore structure) that can be manipulated to achieve the desired surface and bulk properties by modifying the sol-gel chemistry [21–25]. An important advantage that crosslinked silica aerogels offer that is lacking in other commonly used biomedical and biological materials, is the ability to process the aerogels for circuit design and development. This means that “smart” aerogel implants potentially can be designed specially, for neuronal stimulation and guidance and this will be investigated in future studies by the authors.

Past studies have focused on investigating the effect of porosity on cell response, and separately, substrate stiffness. Here the authors investigated the combined effect because of the nature of aerogels. For these investigations, PC12 pheochromocytoma cells were used because they represent a well characterized model to study neural differentiation and in particular neurite extension in response to environmental cues [28–29]. Moreover, nerve growth factor (NGF)-treated PC12 cells exhibit features of mature terminally differentiated sympathetic neurons and are electrically excitable [30]. Recent studies have also demonstrated that surface modulus and microtopography affect the extension of neurites by PC12 cells [31]. Aerogels have unique surface properties compared to materials traditionally used in cell culture applications [32, 33] and are the focus of this study. Unlike native aerogels, crosslinked aerogels can be exposed to certain types of solvents and can tolerate chemical treatments which would make it possible to create electrical circuitry intended for nerve guidance and stimulation on these materials. Published work has shown the suitability of crosslinked silica aerogels both for *in vitro* and *in vivo* investigations [21–25].

The aim of this research was to (a) establish the methodology and processing techniques for culturing of PC12 cells on polyurea crosslinked silica aerogels (PCSA), (b) assess the integrity of the aerogel substrate after exposure to culture and fixing conditions and, (c) characterize and examine the effects of PCSA substrate on neurites. Here we stimulated cells with NGF, a growth factor essential for the survival, differentiation and functional activities of neurons in the peripheral and central nervous system. Three different substrates were studied: (1) PCSA, (2) PDMS, and (3) TC plastic. Neurite length, cell cluster size, and cell morphology and arrangement were compared between the different surfaces. The cell adhesion and distribution was also observed by the morphologic method after fluorescent staining.

## Materials and methods

### Synthesis and preparation of aerogel and PDMS substrates

For aerogel synthesis, 8.75 mL methanol was added to 1.5 mL deionized (DI) water. To the methanol and DI water mixture, 3.85 mL tetramethyl orthosilicate (TMOS) (Sigma-Aldrich) and 0.25 mL (3-aminopropyl) triethoxysilane (APTES) (Sigma-Aldrich) were added simultaneously. The mixture was then stirred for 15 sec after which the mixture was poured into custom-designed molds of 5 cm diameter and allowed to cure. After curing, samples were allowed to sit in methanol (Sigma-Aldrich) for 12–24 hrs. Samples were then subjected to a 4 day acetonitrile bath with acetonitrile exchanges every 24 hrs. To cross-link the samples, they were transferred to a 33gr Desmodur N3200 (Bayer Material Science)/94ml acetonitrile (Sigma-Aldrich) mixture and allowed to soak for 24 hrs. Samples were then transferred back to pure acetonitrile and allowed to sit in a 70°C oven for 3 days. Samples were then subjected to 4 more days of acetonitrile baths with exchanges every 24 hrs. Samples were then dried by critical point drying as discussed previously [21, 22]. PDMS (Sylgard 184, Dow Corning) samples were also prepared with a 10:1 polymer to crosslinker) ratio, described in detail previously [23]. Aerogel and PDMS samples were cut into 5x5mm^2^ coupons prior to sterilizing and coating with Matrigel.

### Cell preparation and culture

#### PC12-C41 cells culture conditions

A clone of rat PC12 pheochromocytoma cells [34], PC12-C41 was obtained from CH3 BioSystems LLC. PC12-C41 cells were cultured in RPMI 1640 with GlutaMAX (Life Technologies) supplemented with 10% heat-inactivated horse serum,5% fetal bovine serum, and 50μg/ml penicillin/streptomycin (complete medium). Medium was changed every 2–3 days while the cultures were maintained in a 5% CO_2_ incubator at 37°C. Prior to experiments, cells were “primed” for neurite extension by replacing the complete medium with RPMI 1640 supplemented with 50 ng/ml NGF (EMD Millipore) and 1% heat-inactivated horse serum for 14 days. Cell seeding densities of 1x10^4^ cells/cm^2^ to 5x10^4^ cells/cm^2^ were tested on Matrigel (Becton Dickinson/Corning). A final seeding density of 1x10^4^ cells/cm^2^ was found to give optimal results meaning that this density allowed for a spacing between cells and cell clusters which was conducive for neurite length measurements. Cells were seeded onto Matrigel coated aerogel disks, PDMS, and 35mm TC plastic petri dishes (control) with 50ng/ml NGF. Cell growth and process development were carefully monitored by means of a Nikon Eclipse TS100 microscope for 24 hrs before fixing and staining for fluorescence microscopy or scanning electron microscopy.

#### PC12 cells culture conditions

For the remainder of the study, PC12 cells acquired from ATCC were used (explanation in Results section). Cells were cultured in complete medium under the same conditions as explained for PC12-C41. PC12 cells were “primed” with NGF for 8 days on collagen coated TC plastic in low serum medium before experiments. For experiments, cells were plated at a density of 1–1.5 X 10^4^ cells/cm^2^ which was discovered to give optimum results.

#### Matrigel and collagen coating of cell substrates

All the procedures were performed in a sterile tissue culture hood. Prior to coating with either Matrigel or collagen 1, PCSA, PDMS, and TC plastic substrates were sterilized by a 10 sec submersion in ethanol followed by exposure to ultraviolet (UV) lamp in the culture hood for 1 hr [21].

#### Matrigel coating

Matrigel (Becton Dickinson/Corning) was thawed on ice in a 4°C refrigerator before being diluted to a concentration of 1 mg/ml in serum-free RPMI 1640 while still on ice. Using pre-cooled pipettes, the diluted Matrigel solution was pipetted onto the substrate and spread manually with a pipette tip to cover the area using about 1μl of Matrigel to cover 1 mm^2^ of substrate area. This allowed us to achieve a protein density of 100μg/cm^2^ as recommended by the manufacturer. After a 1 hr incubation time at room temperature, the unbound material was aspirated with a pipette connected to a vacuum line and the substrates were washed with serum-free RPMI1640 once. The samples were kept submerged in RPMI1640 until used.

#### Collagen coating

Rat tail collagen (Invitrogen) was diluted to 0.05 mg/ml in 20 mM acetic acid, per manufacturer recommendations. Sufficient collagen was then added to samples to acquire a protein density of 4 μg/cm^2^ [35] and was incubated for 1 hr at room temperature. The unbound material was aspirated with a pipette connected to a vacuum line and the substrate were washed twice with serum free RPMI 1640.

### Fluorescence staining and confocal scanning fluorescence microscopy

Cells and substrates were fixed for 5 min in a 4% formaldehyde (Tousimis) in phosphate buffered saline (PBS) followed by three 5 min washes in PBS. Cells were then permeabilized with 0.1% NP-40 (EMD Millipore) in PBS followed by a 5 min wash in PBS. The samples were then incubated for 30 min at room temperature with phalloidin conjugated to Alexa Fluor 488 or 546 (Molecular probes Inc.) diluted 1:100 in PBS. After three 5 min washes in PBS, samples were mounted on a glass coverslip with the mounting medium Prolong Diamond containing the nuclear stain DAPI (Life Technologies). For cells plated on 35 mm in diameter polystyrene dish, the cells were fixed and stained in the dish. The bottom of the dish was then cut out with a hot spatula and the cells were mounted on a cover glass with Prolong Diamond with DAPI and allowed to sit at room temperature for 24 hrs or until completely dry.

Imaging was performed with a Nikon A1 confocal microscope. The total area analyzed for each sample was about 25 mm^2^. A 5x5 mm^2^ grid was drawn onto the polystyrene sample from which a block was randomly selected for imaging. The three different substrates were systematically imaged by raster-scanning until the entire 5x5 mm^2^ sections were imaged. For all fluorescence images, either a 20X objective/0.75 numerical aperture (NA) or 40X/1.3 NA was used. Data was then collected from each image individually and were analyzed using Image J software.

### Scanning electron microscopy (SEM)

Cells on their substrate were fixed for 2 hrs with 2.5% glutaraldehyde in 0.1M sodium cacodylate buffer. Next, 2x10 min washes in 0.1 M sodium cacodylate buffer were performed. Samples were then immersed in 1% osmium tetroxide for 1 hr followed by 2x10 min washes in 0.1 M sodium cacodylate buffer. Samples were then dehydrated by a series of 10 min washes in 10%, 30%, 50%, 70%, 90%, and 100% ethanol. Samples were then allowed to air-dry and were then sputter-coated with a 10 nm layer of gold. Cells on their substrate were then mounted on a stub using silver adhesive tape. Imaging was performed with a Nova NanoSEM 650 Field Emission Scanning Electron Microscope (FEI Co.).

### Measurements of neurite length and cell cluster area

Neurite lengths and cell cluster areas were measured using NIH Image J software. Only processes greater than two cell soma lengths (14 μm) were considered as neurites [36]. Neurites were traced using a manual length measuring tool. Cell cluster area of the neurites was found by tracing the cell clusters in the plane of the neurites using an area tool. Neurite bearing single cells have also been included in the cluster area graphs. Clusters were defined as consisting of cells in close proximity such that they contact each other and such that a definitive perimeter for the individual cells cannot be determined. Therefore, when determining the area information for the associated neurites, the perimeter of the cluster is traced. The perimeter of the cluster was used to calculate area information for each cluster.

### Evaluation of substrate stiffness

Aerogel disks of 5 mm in diameter were sterilized as described above, incubated in culture medium for 24 hrs, and underwent all the processing steps carried out for cell plating. The disks were then removed from culture medium and stiffness measurement was performed immediately thereafter to prevent the substrates from drying. For the measurement, the substrates were placed on the stage of a Mark 10 ESM 303 tensile tester. Using a “wedge “extension a compression test was performed at a rate of 0.5mm/min with a 20 lb BG series force gauge and the stiffness of the substrate was compared to a control piece that was never exposed to culture medium. Measurement was stopped prior to overloading of the gauge.

## Results and discussion

### Adhesion and growth of PC12 cells on various substrates

**Fig 1:**
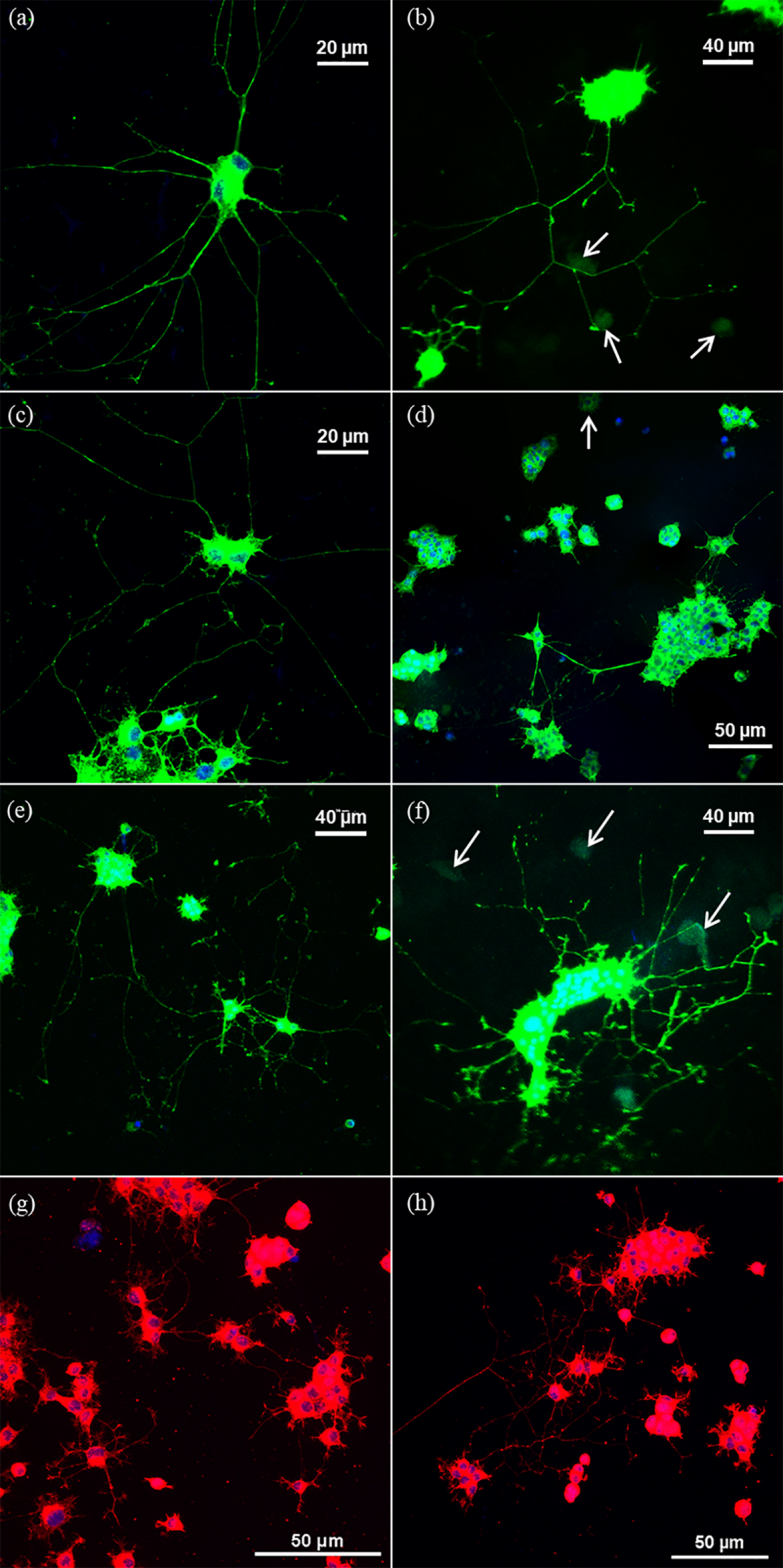
Fluorescence microscopy of PC12-C41 cells. Actin and nucleus stained confocal microscope images of PC12-C41 cells on a Matrigel-coated PDMS (a), (c), (e) and Matrigel-coated aerogels (b), (d), (f) at various magnifications. Here, arrows indicate cells lying on lower planes and are out of focus. PC12-C41 cells were also cultured on tissue culture plastic (control) for comparison and are shown in (g) and (h).

We were able to successfully culture and image PC12-C41 and PC12-ATCC cells plated on PDMS, aerogel, and TC plastic Matrigel-coated substrates. Cells were imaged by confocal fluorescence microscopy after staining with the fluorescent dyes DAPI, to stain nuclear DNA, and phalloidin conjugated to an AlexaFluor^®^ dye to stain actin filaments and outline the overall cell shape. Fig 1 shows examples at various magnifications of such imaging experiments for PC12-C41 cells plated on Matrigel-coated PDMS, aerogel, and TC plastic. Extensive neurite outgrowth and connectivity can be observed for all substrates, irrespective of their surface topography. Fig 1a, 1c and 1e show the adhesion and extension of processes on smooth PDMS substrates collected from various locations of the PDMS samples. The cellular activity resembles the behaviors observed on smooth cell culture plastic. Fig 1b, 1d and 1f show the behavior of PC12-C41 cells on polyurea crosslinked silica aerogel substrates, collected randomly from various locations of the substrates and are representatives of the cell behavior on this substrate. In the case of aerogels, due to the 3-D nature of the surface, cell adhesion and proliferation occurred on multiple planes as indicated by the white arrows in Fig 1 which show cell clusters on focal planes other than the one that microscope was focused on. The 3-D distribution of the cells and their extensions make imaging challenging and the images in Fig 1 show only examples of one particular focal plane. These images were captured intentionally with laser intensities high enough to reveal the weak actin staining in the processes. Under these conditions, the cell processes which are richer in actin than the cell bodies appear saturated in intensity. The behavior of PC12-C41 cells on TC plastic (control) is shown in Fig 1g and 1h for comparison. Regardless of the substrate morphology and topography, PC12-C41 cells consistently formed large clusters on all 3 substrates (TC plastic, aerogel, and PDMS) (discussed further below) and consequently made neurite length calculations complicated. Rat tail collagen pre-coated cover-slips (Becton Dickinson) were also tested during the early stages of this study, however, they did not provide adequate protein density for PC12 adhesion and extension of processes. Matrigel was chosen instead for its mix of extracellular matrix proteins that better mimics conditions *in vivo* [37].

**Fig 2:**
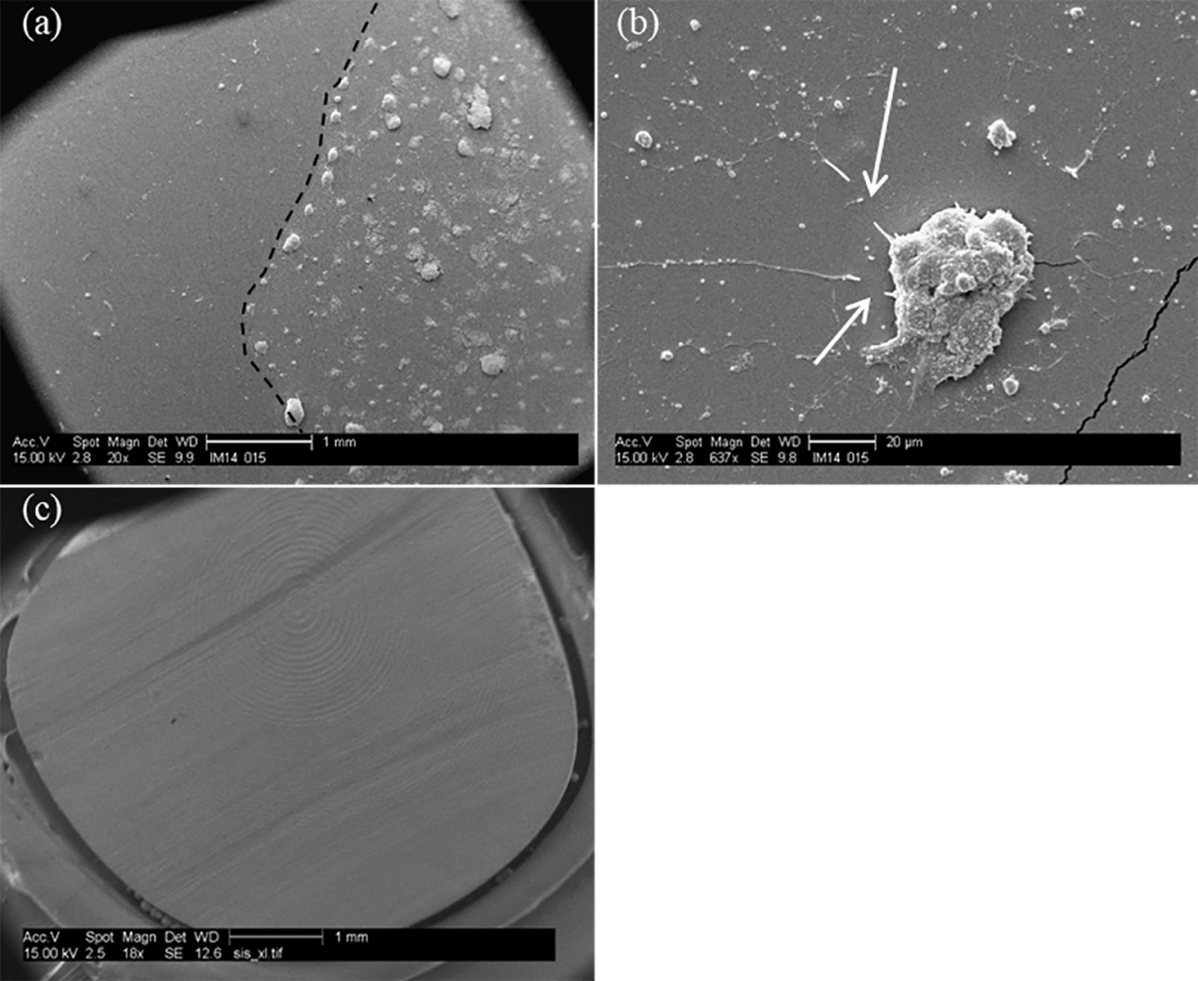
Controlled adhesion of PC-12C41 on substrates. SEM images of the boundary between Matrigel-coated and uncoated regions of PDMS (a) and (b) and an aerogel substrate (c), in the absence of any Matrigel coating. Dotted line outlines the boundary with the two sections and, the arrows indicate broken processes as a result of handling and fixing protocol implemented.

A Matrigel layer proved to be necessary for the adhesion and proliferation of cells on all three substrates of Fig 1, as evidenced by the SEM images in Fig 2. Fig 2a shows the boundary between Matrigel-coated and uncoated areas (white dotted line) on a PDMS substrate where the adhesion and proliferation of cells only occur on the Matrigel-coated region and therefore allow for regional control of growth and patterning of these substrates if desired. In Fig 2b a closer view of neurites on the Matrigel-coated PDMS is shown and the white arrows indicate break in the extensions as a result of fixing and handling protocols for SEM imaging. Similarly, without the Matrigel layer, no cells adhered to the aerogel substrate, as evidenced by Fig 2c once again demonstrating the potential for patterning of these substrates and guidance of cell growth. Similar behavior was observed on plastic substrates (images not shown). These results are consistent with previous findings with dorsal root ganglia cells cultured on aerogels [21, 24] where an extracellular matrix protein adhesion layer was found to be necessary for adhesion and proliferation of these cells on aerogel substrates. The importance and significance of this observation is the control and nerve guidance that one can accomplish by means of coatings such as Matrigel on such substrates.

### ATCC versus C41 cells

In this study, differences were also noticed between PC12 parental cells and PC12-C41 clone [34] with respect to neurite extension with cell plated on the different substrates described above. The percentage of cells extending neurites was lower for PC12-C41 relative to PC12 parental cells. Moreover, it took longer for PC12-C41 cells to extend neurites relative to PC12 parental cells. The majority of PC12-C41 cells would not extend neurites even in the presence of NGF, which increased only their rate of proliferation. Side by side comparison of PC12-C41 with PC12 from ATCC showed that after exposure to NGF, PC12 differentiated much more rapidly and produced much longer neurites than PC12-C41.

**Fig 3:**
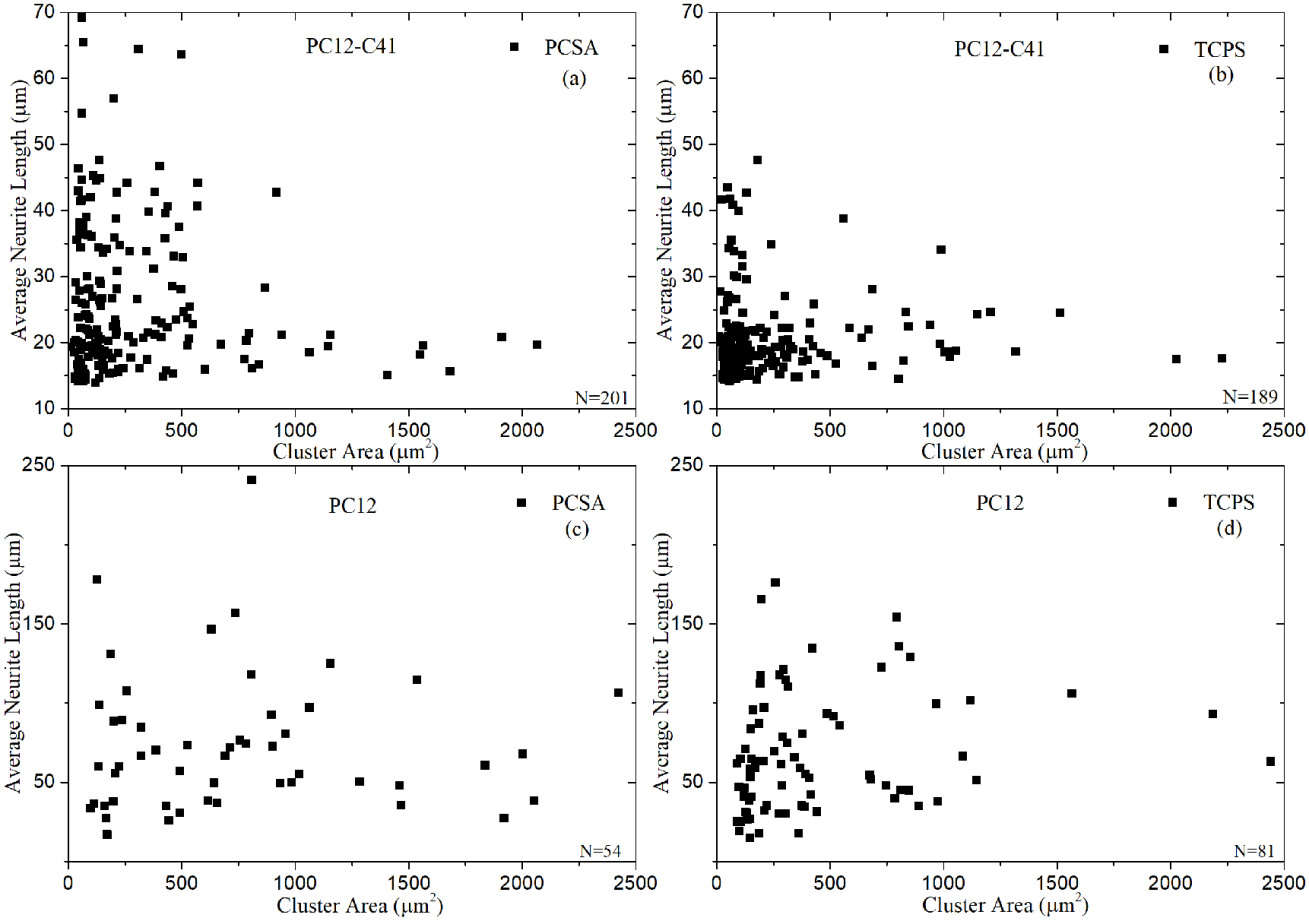
Neurite length and cluster size; PC12- C41 versus ATCC. PC12-C41 cells formed very large clusters on (a) aerogels and (b) TC plastic with the average neurite length decreasing as the cluster size grew. ATCC cells however, formed substantially smaller clusters with much longer average neurites on (c) aerogel and (d) TC plastic substrates.

In addition, a much higher percentage of PC12 differentiated than PC12-C41 as determined by the extent of neurite extension. In Fig 3 we have compared the average neurite length per cluster area of the C 41 cells (3a and 3b), with the ATCC cells (3c and 3b), for the two different substrates, aerogels and control with a sample number of n = 2. Results show that C41 cells formed larger clusters on both aerogel and TC plastic substrates when compared with cluster sizes of the PC12- ATCC cells on similar substrates. PC12-ATCC cells however extended longer neurites on both substrates. For these reasons, we switched to PC12 cells from ATCC for the remainder of the study in order to understand the affect of the substrates on neuron behavior.

### Matrigel and collagen distribution on aerogels

**Fig 4:**
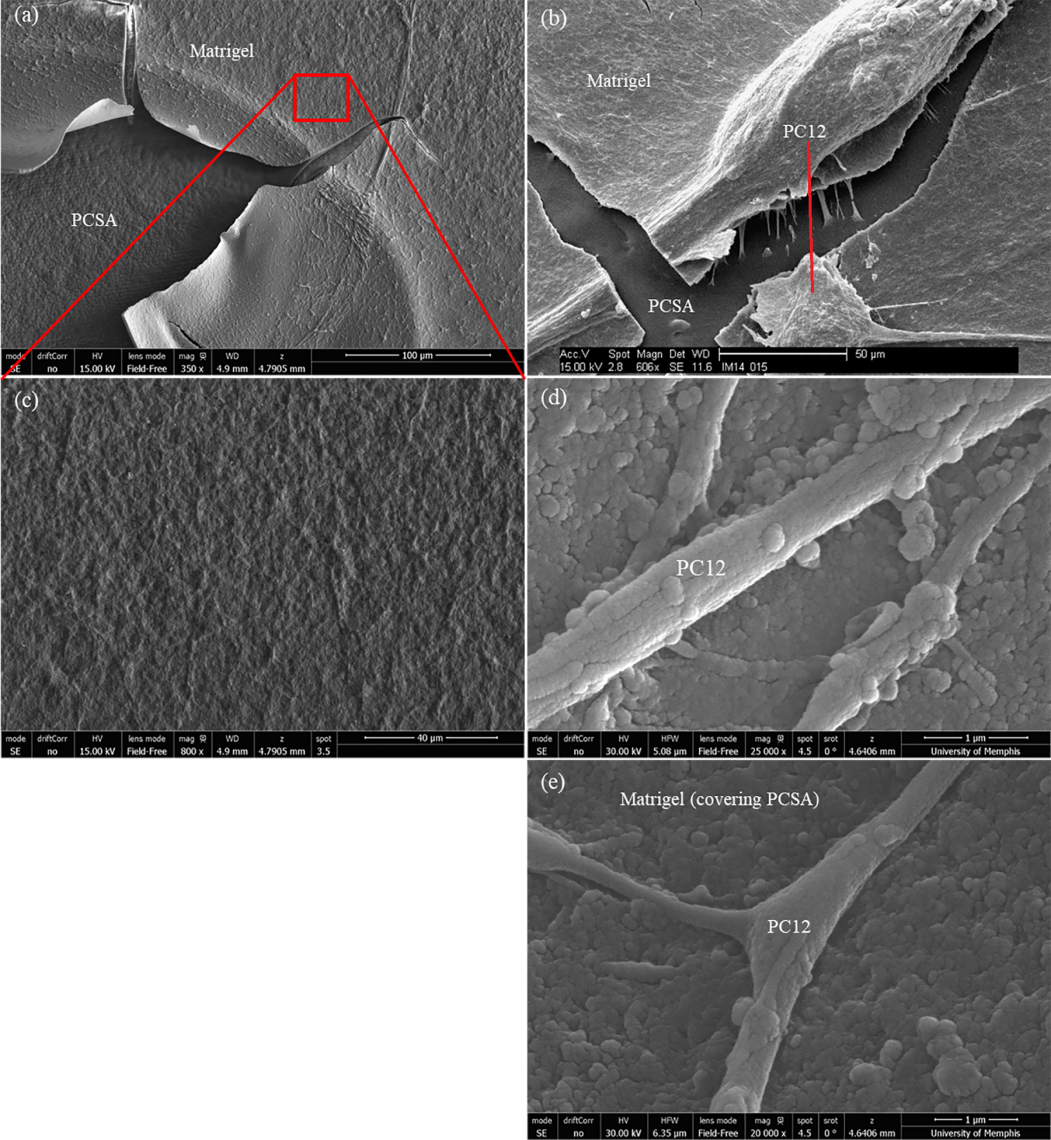
Matrigel on aerogel substrates. SEM images of Matrigel deposited on PCSA and prepped for SEM imaging. (a) Arrows indicate the Matrigel layer peeling away from the aerogel substrate, at the edge of the coupon. (b)PC12+ Matrigel +PCSA, the thickness of the Matrigel layer estimated to be 2–3 μm. (c) Dense fibrous weaves of Matrigel observed on the PCSA substrate. (d) and (e) PC12+ Matrigel +PCSA showing the texture and morphology at the nerve substrate interface.

Fig 4 shows the distribution of Matrigel on aerogel substrates and details of its texture. Fig 4a and 4b were taken from the edge of the Matrigel coating where it had begun to peel away from the substrate which was an artifact of the fixing process that is needed prior to the SEM step. The dense fibrous “weave” of the Matrigel can be seen and the thickness of the Matrigel is estimated to be of the order of 2–4 μm, inferred from the SEM images. Fig 4c shows a higher magnification image of the Matrigel texture. Fig 4d and 4e demonstrate the surface roughness and texture of the Matrigel coating on aerogel that appears to be conforming to the 3-D structure of the surface of the aerogel underneath it, to a degree. Up-close (4d and 4e) the Matrigel layer on aerogel shows a highly textured 3-D morphology that was not seen on tissue culture plastic preps and appears to be unique to the way that Matrigel distributes itself on the aerogel substrate.

**Fig 5:**
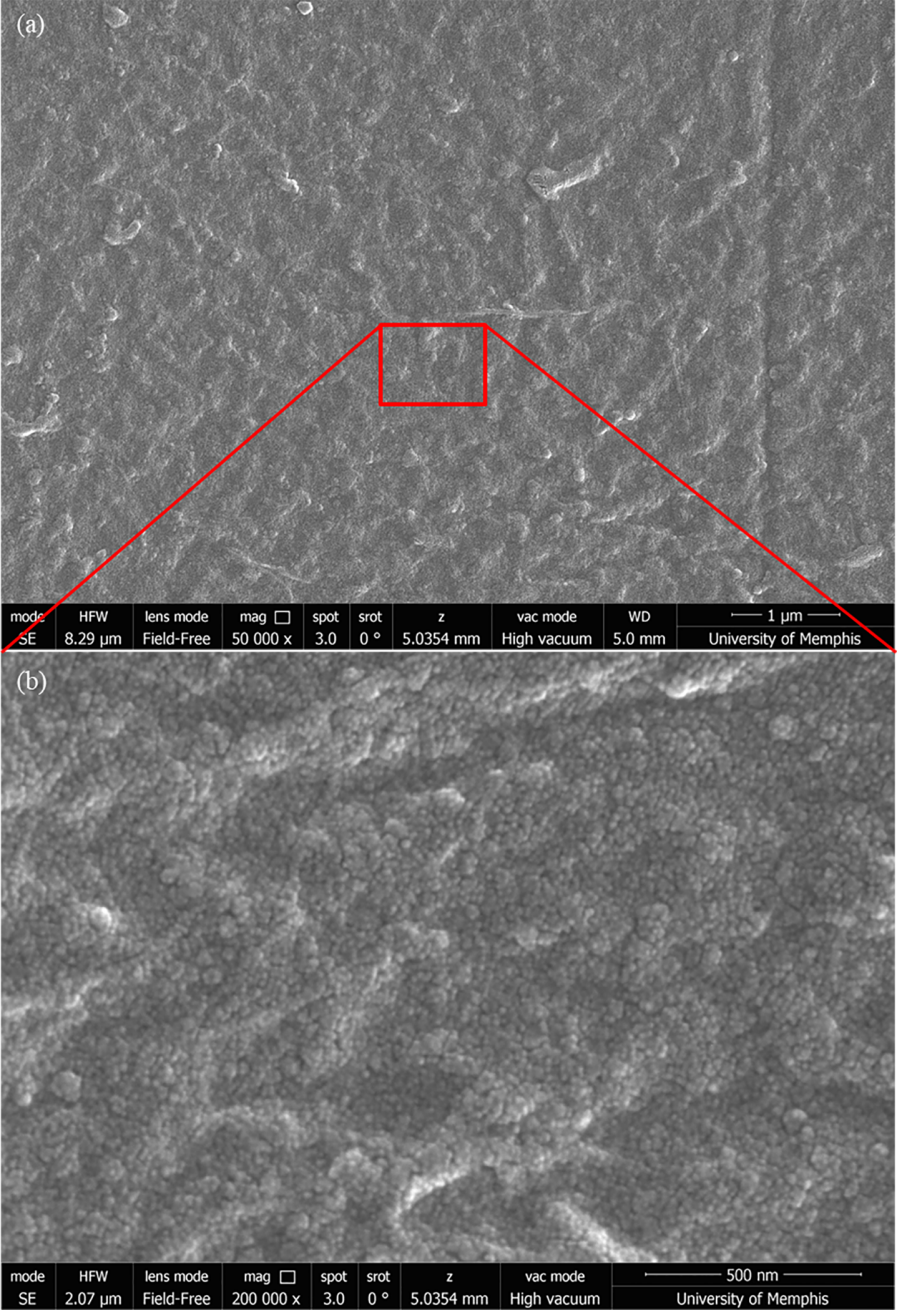
SEM image of collagen on aerogel. SEM images of collagen deposited on an aerogel substrate showing a continuous layer of coating (a) that still appears to have the surface topography of the aerogel surface underneath intact (b).

**Fig 6:**
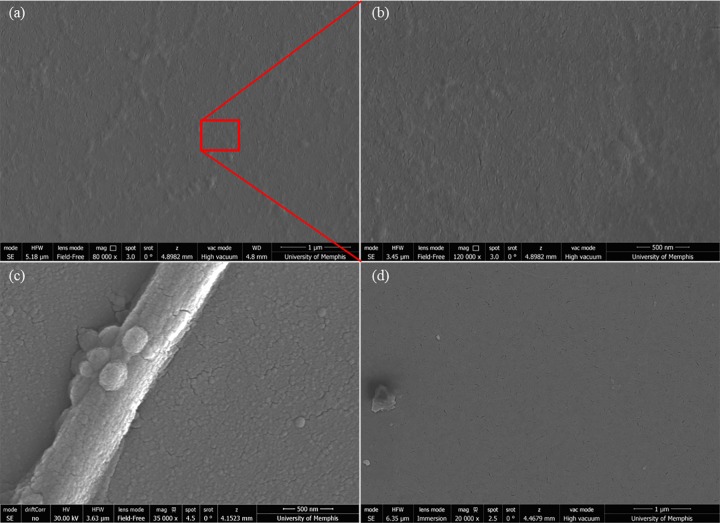
SEM images of collagen and Matrigel on TC plastic. Matrigel coating on TC plastic shows a continuous but planar layer as shown in (a) and (c) with a dense network of fibers. Collagen coating on TC plastic also appears continuous and somewhat planar, (b) and (d), but with a more granular structure than Matrigel.

SEM images of collagen-coated aerogels are shown in Fig 5. Fig 5a shows a continuous and dense collagen layer that does not show a fibrous structure. Fig 5b shows a higher magnification of the region in 5a and a 3-D topography can be seen. It is believed that this topography is influenced by the aerogel substrate underneath. The distribution and texture of Matrigel and collagen on TC plastic was also investigated and served as control and imaged by means of scanning electron microscopy. Fig 6 shows the distribution of Matrigel (6a and 6b) and collagen (6c and 6d) on TC plastic and while the texture appears to be similar to that observed on aerogels, it is planar in comparison. It is clear from these images that the coating, Matrigel or collagen, clearly conforms to the substrate topography and therefore transfers the substrate “texture” to the cells to some extent.

### Effect of aerogel substrate on PC12 behavior

**Fig 7:**
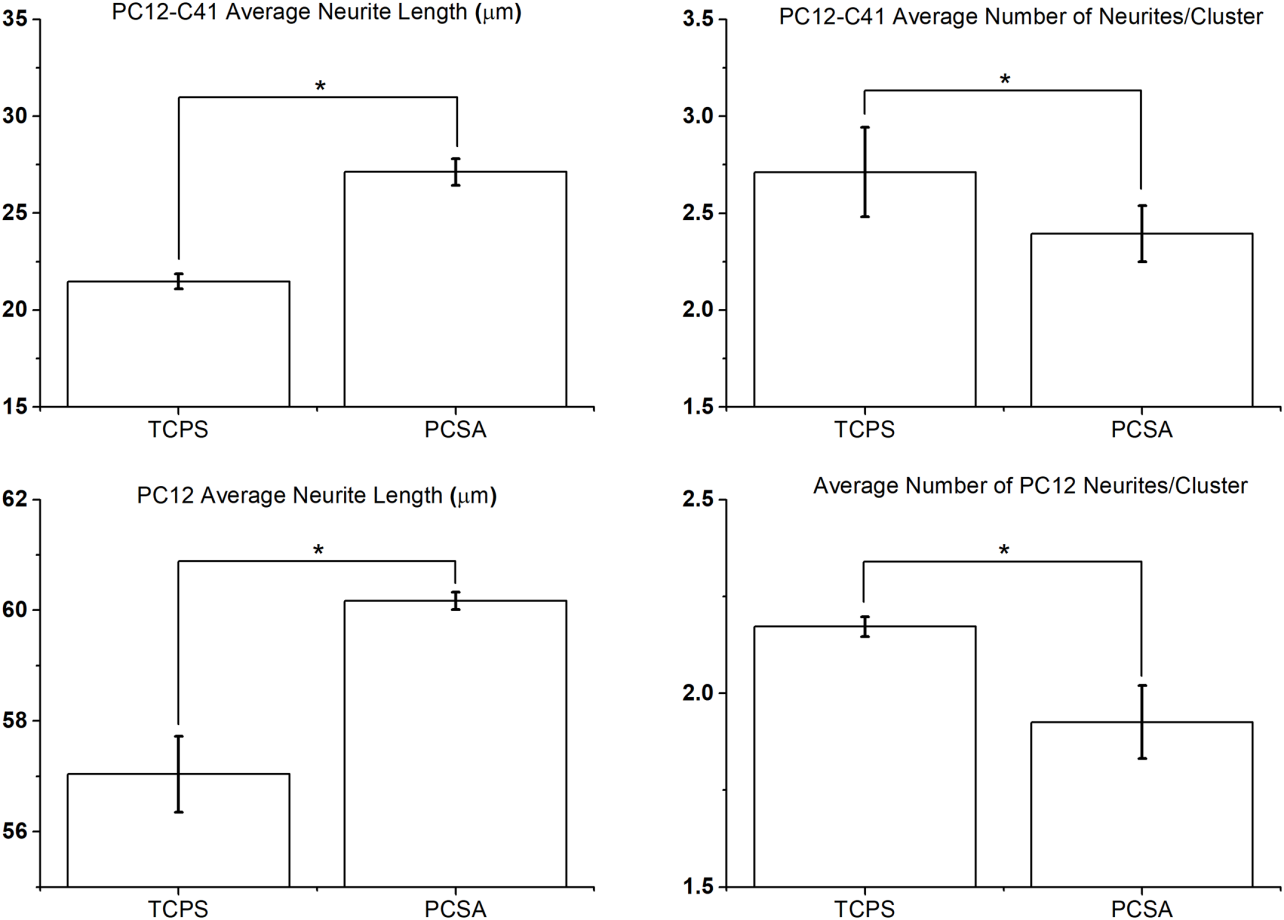
Affect of aerogel substrate on neurite response. Average neurite length for PC12 -C 41 and PC12-ATCC cells are longer on aerogel substrates compared to TC plastic; (a) and (c). (b) The average number of neurites per cluster area however, is lower on aerogels than on TC plastic (control) as seen in (b) and (d) for both PC12-C41 cells and ATCC cells. Error bars represent standard error of mean and sample size n = 3, * indicates significance with p<0.05, obtained from a student's t-test.

No noticeable differences of the cell morphology and the cell shape were observed on the different substrates. The average measured neurite length on PCSA was longer than the average neurite length measured on TC plastic, for both C41 and ATCC cells. The number of neurites per cluster however was lower on PCSA than on TC plastic, again for both PC12 types as shown in Fig 7. In all cases error bars represent standard error of mean and represent data averaged from three separate trials n = 3. The * symbol on Figs 7a, 7b, 7c and 7d indicate significance with p<0.05, obtained from a student's t-test. Presented results suggest that aerogels support extension of longer neurites, with fewer number of extensions. Similar results were observed previously [38,39] where stochastic surface nano-roughness clearly modulated PC12 response and depended on the roughness scale.

**Fig 8:**
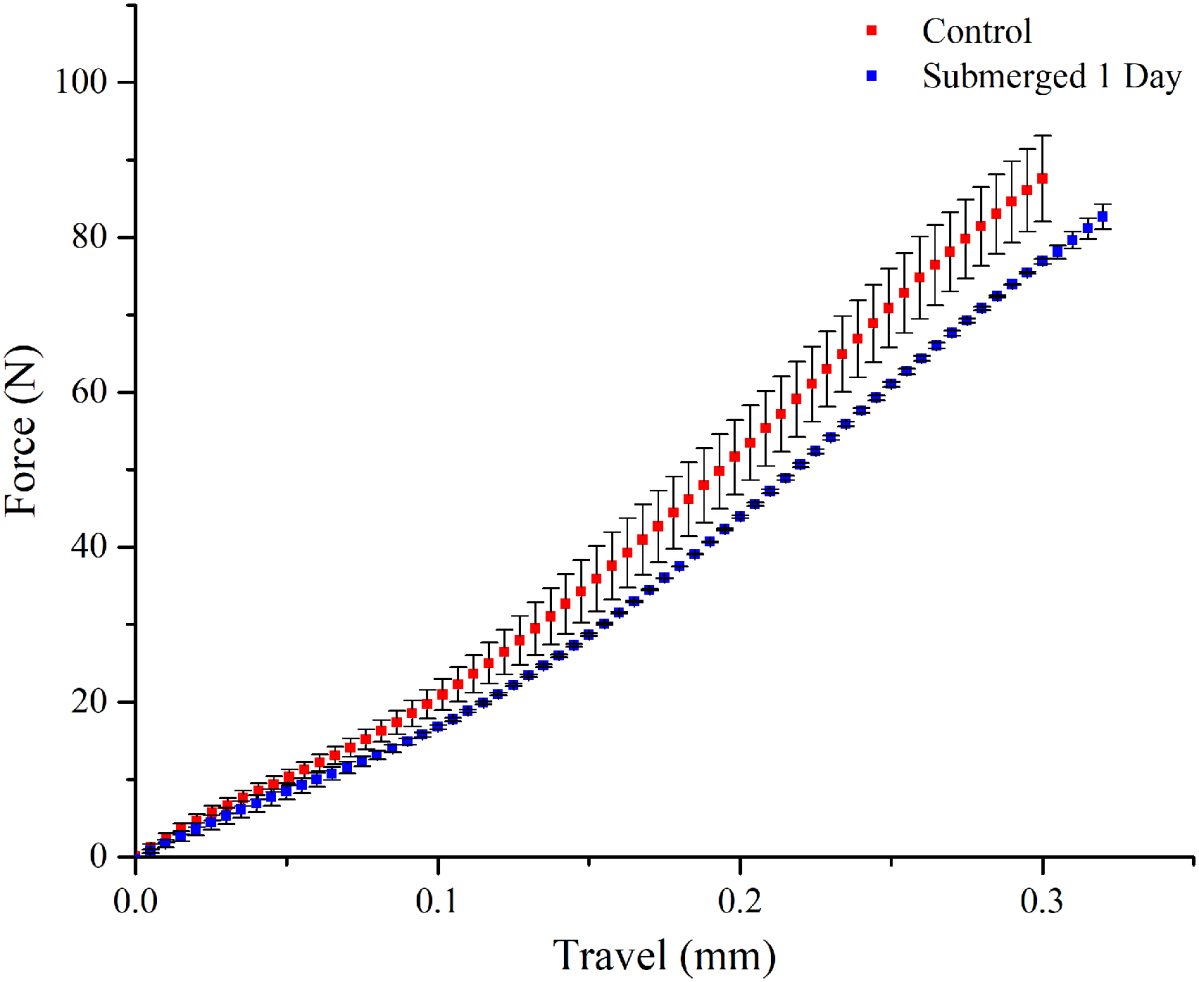
Substrate stiffness. Compression studies performed at a rate of 0.5 mm/min on the control (dry) and incubated aerogel samples showed a change in the surface stiffness up to a depth of 0.3 mm. Sample size n = 3.

Given that substrate stiffness plays an important role in the neuronal response, we investigated the integrity of the aerogel’s mechanical integrity while incubated in cell culture medium. Compression studies performed at a rate of 0.5 mm/min on the control (dry) and incubated aerogel samples showed a change in the surface stiffness up to a depth of 0.3 mm, after sterilization and incubation in cell culture medium, as shown in Fig 8, averaged over multiple measurements, n = 3 where error bars reflect standard error of the mean. Exposure to UV followed by incubation in cell culture medium lead to a slight “softening” of the outer-most regions and surfaces of the aerogels, tested to a depth of 0.3 mm. Therefore, the cells are in fact responding to a slightly “softer” PCSA, that is still considered stiff in comparison to cell culture plastic and PDMS substrates as mentioned in references [32, 33, 40].
